# Education Research: Brick by NeuroBrick—A Collaborative Framework for Digital Content Creation

**DOI:** 10.1212/NE9.0000000000200278

**Published:** 2025-12-10

**Authors:** Ashley Paul, Natalie Vallejo, Nana Boakye Agyeman Badu-Prempeh Badu-Prempeh, Rachel Marie E. Salas, Doris G. Leung, Tao Le, Tamara Kaplan

**Affiliations:** 1 Department of Neurology, School of Medicine, Johns Hopkins University, Baltimore, MD;; 2 ScholarRx, Elizabethtown, KY;; 3 Department of Medicine, School of Medicine, University of Louisville, KY;; 4 Department of Neurology, Mass General Brigham, Boston, MA; and; 5 Harvard Medical School, Boston, MA.

## Abstract

**Background and Objectives:**

Medical students increasingly rely on online resources, many of which are unvetted or behind paywalls, creating variability in quality and access. To address this, Johns Hopkins University School of Medicine (JHUSOM) and Harvard Medical School (HMS) developed NeuroBricks—peer-reviewed, case-based neurology modules designed to strengthen clinical reasoning and decision making. This study evaluated their feasibility, effectiveness, and scalability by examining student satisfaction and perceived educational impact across 2 institutions with different curricular structures.

**Methods:**

We conducted a retrospective feasibility and evaluation study of the NeuroBricks Library, analyzing anonymous survey data from medical students at JHUSOM and HMS collected between January 2023 and June 2025. At JHUSOM, NeuroBricks were required during the neurology clerkship; at HMS, they were offered as optional resources. Surveys assessed satisfaction, perceived improvements in clinical skills, and preferred learning modalities. Five pilot NeuroBricks were initially released, expanding to 13 by November 2024. Likert scale responses were analyzed descriptively, and open-ended comments underwent thematic analysis. We also collected informal feedback on participant perceptions of the NeuroBrick creation process, along with anonymized platform analytics on global reach and usage.

**Results:**

A total of 343 medical students participated. At JHUSOM, mean satisfaction ratings for the 5 pilot NeuroBricks ranged from 4.08 to 4.11 (5-point scale). Among 71 students evaluating the complete 13-module library, most reported improved recognition of neurologic symptoms (54 [76.1%]), interpretation of abnormal findings (48 [67.6%]), and preparedness for patient encounters (52 [73.2%]). Most preferred asynchronous learning (49 [69%]) and rated interactive features as engaging (48 [67.6%]). At HMS, 85% (17/20) were likely to recommend NeuroBricks, with thematic analysis revealing increased confidence, targeted learning, and supplemental value for shelf examination preparation. Module developers reported positive perceptions of the creation process, and analytics demonstrated global reach across 34 countries.

**Discussion:**

The multi-tiered mentorship model successfully supported the creation of the NeuroBricks Library while students demonstrated strong satisfaction with its content and perceived impact. Required integration into clerkships promoted greater engagement than optional use. By providing peer-reviewed, clinically focused modules, this scalable, open-access approach helps overcome key barriers in neurology education and can be extended to other specialties, particularly where faculty or resources are limited.

## Introduction

Over the past decade, medical education has increasingly shifted toward online, virtual, and asynchronous formats. Some experts suggest that we may now envision a future where medical education is delivered exclusively online, offering global access to the world's leading professors and standardized assessments.^1,2^ The effectiveness of virtual education became especially apparent during the COVID-19 pandemic, when learners could no longer safely gather for traditional lectures or bedside training.^3-5^ Learners and educators turned to digital platforms out of necessity—and found unexpected advantages, including cost-effective delivery and access to a broader range of topics than typically available at any institution.^6,7^

However, this shift has revealed new challenges. Medical students are increasingly supplementing their education with unvetted resources such as YouTube, Instagram, TikTok, and, more recently, large language models (LLMs)—platforms that are easily accessible but often lack quality control.^8,9^ Third-party commercial resources, particularly those focused on the United States Medical Licensing Examination (USMLE) preparation, have also proliferated—sometimes at the expense of patient-centered education. Meanwhile, expert-reviewed content is often hidden behind paywalls, contributing to inequities in access. Even when educators strive to create high-quality materials, they encounter significant time constraints due to competing demands, including clinical duties, faculty development, accreditation requirements, and student assessment.^10,11^ As a result, educational efforts are often duplicated across institutions, with limited coordination or sharing—leading to siloed content development and inefficient use of scarce faculty expertise.^12^ The field urgently needs a scalable method for producing accurate, peer-reviewed, and accessible educational content that aligns with the self-directed learning styles of today's digital-native students.

The NeuroBricks Library was created as a collaborative initiative between Johns Hopkins University School of Medicine (JHUSOM) and Harvard Medical School (HMS) to address the evolving challenges in neurology education. These concise, peer-reviewed, case-based modules complement traditional clerkship teaching, improving educational quality, accessibility, and equity. Recognizing the importance of sustainable content creation, this initiative uses a multi-tiered mentorship model supported by an interinstitutional editorial review board. This innovative approach enables rapid development of validated learning materials without compromising quality. It also distributes the workload, fosters cross-institutional collaboration, and provides meaningful scholarly opportunities for both students and faculty.

The goals of this study were to evaluate whether low-fidelity but high-quality, peer-reviewed online modules in neurologic education for medical students could be developed in a way that minimizes the content creation burden for individual faculty. We also aimed to determine whether these modules would be well received as a valuable educational tool that supports students' perceived development of clinical skills relevant to caring for patients with neurologic diseases.

## Methods

### Curriculum Planning and Platform Selection (January–March 2021)

The NeuroBricks' general learning objectives were aligned with national neurology curriculum guidelines and aimed at enhancing students' clinical reasoning skills.^13^ Topics were chosen according to these guidelines, input from medical students who had completed the Neurology Core Clerkship, and observations from clerkship directors about common areas where students face difficulties.

The ScholarRx platform was selected for this initiative because it provided a user-friendly interface, adaptability, and support for open-access content creation. It allowed authors to develop short, modular, interactive learning experiences that could be created de novo or adapted from existing content. The platform did not require advanced technical expertise, reducing training needs and time investment. Features such as “cloning” enabled educators at different institutions to duplicate and modify existing modules for their local learning environments while asynchronous editing supported cross-institutional collaboration. Materials were distributed under a Creative Commons license, ensuring broad accessibility without subscription requirements. ScholarRx was chosen for these perceived advantages; however, the experience was not formally compared with other authoring platforms.

### Implementation of Standard ScholarRx Modules (2021–2022)

In March 2021, standard ScholarRx-developed modules covering key neurology topics were introduced into the preclerkship curriculum of the Neurology Core Clerkship at Johns Hopkins. Between March 2021 and March 2022, 85 students engaged with these modules and evaluated them through anonymous online surveys. Initial feedback indicated that, although students found the modules helpful, they were overly focused on pathophysiology—a focus more appropriate for the preclinical years. Students expressed a preference for clinically oriented modules during the clerkship.

### Development of the First NeuroBrick and Adoption of the Clinical NeuroBricks Formula (October 2022–March 2023)

In response, a medical student and faculty developed a NeuroBrick entitled “Approach to Tremor” (later renamed “I Have a Tremor”), which was launched in October 2022. This NeuroBrick was designed to be clinically focused and aligned with the learning objectives of neurology clerkship students. Immediate feedback from 14 students^14^ who piloted the first NeuroBrick informed a new development approach, ensuring that future NeuroBricks would target clinical reasoning skills relevant to real-world clinical practice (eTable 1). This model was formally adopted on March 6, 2023; 4 additional NeuroBricks were created using the “Clinical NeuroBricks Formula.”

### Formalization of the Multi-Tiered Mentorship Model (March 2023 Onward)

**Figure 1:**
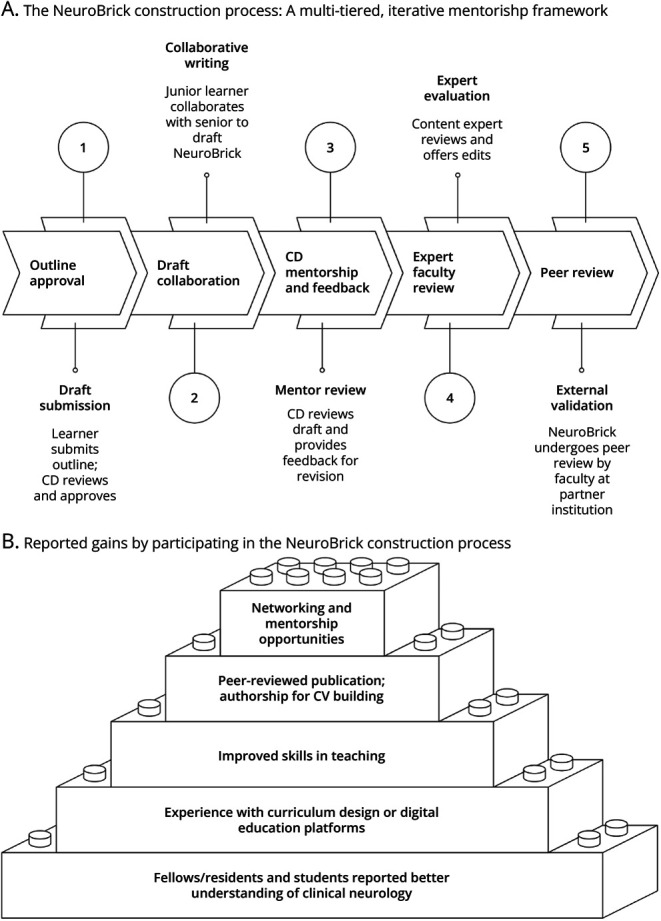
The NeuroBrick Construction Process: A Multi-Tiered, Iterative Mentorship Framework (A) This framework illustrates the stepwise development of educational NeuroBricks through layered mentorship and iterative feedback, supported by a virtual community of practice that fosters cross-institutional collaboration and shared expertise. (B) Among 26 contributors to the NeuroBricks Library, 5 faculty members, 5 residents/fellows, 2 medical students, and 1 undergraduate shared informal reflections through personal correspondence. CD = Clerkship Director.

The process of creating the NeuroBricks Library used a multi-tiered mentorship model and review system to ensure high-quality content while alleviating the burden on individual faculty members (Figure 1A). Rather than relying on a single faculty author, the development team for most NeuroBricks consisted of a medical student, a resident, and a faculty member as the senior author. This collaborative approach aligned with the virtual community of practice (vCoP) social learning theory.^15-17^ This model promoted collaboration across institutions by enabling faculty to share expertise, refine educational materials, and collectively enhance teaching practices. Learners also engaged in this vCoP by contributing to NeuroBrick development, gaining mentorship, and acquiring hands-on experience in medical education scholarship.

Contributors underwent training to become familiar with platform guidelines and learning objectives. Each Brick was initially drafted by medical students and/or residents, using the “Clinical NeuroBricks Formula,” developed by the authors of this article (eTable 1). Drafts were initially reviewed by a neurology resident or fellow, followed by an attending physician to ensure accuracy and pedagogical soundness. To enhance rigor, content validity, and external review, faculty from a partner institution (either JHUSOM or HMS) conducted a peer review of the materials. The faculty authors of this article acted as a final review board to ensure that each Brick aligned with the intended learning objectives and met standards for clarity, relevance, and engagement.

This collaborative process ensured high-quality content while minimizing the burden on individual faculty. It also promoted educational scholarship by positioning medical students (and sometimes undergraduate premedical students) and housestaff as first authors, with faculty serving as senior authors or editors for peer-reviewed, open-access educational modules. Some students received grant funding through the ScholarRx Bricks Builder Curriculum Development Grant, and several presented their work at national medical education conferences.^18-20^

The initiative has since evolved into a formal educational pathway at Johns Hopkins through the Osler Apprentice Program.^21^ This year-long elective offers medical students experiential learning in medical education. Many students identify curricular gaps based on their experiences, and some develop NeuroBricks to address these needs. A parallel program for undergraduate students, known as the PreDoc Program, has enabled some of our undergraduate students to participate, often under the mentorship of a resident.^22^

### Integration of NeuroBricks Into the Neurology Clerkships (January 2023–June 2025)

#### Sampling and Recruitment

At JHUSOM, all second-year, third-year, and fourth-year medical students completing the Neurology Core Clerkship between January 2023 and June 2025 were required to use the NeuroBricks Library as a learning resource. The full library was provided at the start of the rotation, and students were expected to complete a set number of modules within the first 2 weeks. As part of the clerkship, students were also required to complete the standard evaluation survey used across all JHUSOM core clerkships, which included additional questions about how well the NeuroBricks supported the clerkship's educational objectives.

At HMS, the NeuroBricks Library was offered as an optional supplemental resource during the Neurology Clerkship over the same period. All students were given access at the start of the rotation, but both module completion and survey participation were voluntary.

### Data Collection and Analysis

We conducted a retrospective feasibility and evaluation study of the NeuroBricks Library, analyzing anonymous survey data from medical students at JHUSOM and HMS collected between January 2023 and June 2025. In total, 343 second-year, third-year, and fourth-year medical students participated (JHUSOM: 323; HMS: 20). Data were gathered in multiple phases, corresponding to the staggered release of NeuroBrick modules. Additional data included informal survey feedback from NeuroBrick developers on the creation process, along with anonymized platform analytics on global reach and usage. A retrospective analysis was conducted on this anonymized data set.

### Evaluation Process at JHUSOM During the Pilot Phase From January 2023 to October 2024

At the conclusion of their four-week neurology clerkship, JHUSOM medical students completed evaluation surveys regarding their overall clerkship experience, including a specific subset focused on the NeuroBricks.

From January 2023 to October 2024, medical students evaluated 5 pilot NeuroBricks using a 5-point Likert scale. The 5 pilot NeuroBricks included the following:

1. “I Have a Tremor”
2. “I Can't See Out of My Eye”
3. “I Can't Walk, and I Can't Feel My Legs”
4. “I'm Feeling Off Balance”
5. “My Vision Is Not Right, and My Arm Is Tingling”

Students rated their overall satisfaction, the usefulness of NeuroBricks as a clerkship resource, the appropriateness of the tool for meeting clerkship learning objectives, and whether they would recommend NeuroBricks to other students. Responses were summarized using descriptive statistics. Mean ratings were calculated for each NeuroBrick as a measure of student satisfaction. Mean 5-point Likert scale responses were also analyzed for 3 key statements: “I will recommend this resource to other students,” “The NeuroBricks were helpful as a resource during the neurology clerkship,” and “The neurology clerkship provided appropriate learning tools or opportunities for me to attain the learning objectives.” Counts and percentages were reported.

### Evaluation Process at HMS During the Pilot Phase From May 2023 to January 2024

At HMS, student feedback was collected through an optional evaluation survey. The survey included (1) a 4-point Likert scale question, “How likely are you to recommend the resource NeuroBricks to other students?”; (2) a multiselect item asking, “How did you use the NeuroBricks to study during the clerkship? Check all that apply”; and (3) 3 open-ended questions regarding students' experiences. The open-ended response questions included the following:

1. How, if at all, did using NeuroBricks change your confidence and self-efficacy in the content area?
2. How, if at all, will you use the NeuroBricks moving forward?
3. How, if at all, will NeuroBricks change the way you study?

Likert scale responses were summarized as percentages, multiselect responses as counts, and open-ended responses were analyzed thematically using an inductive coding approach. Two investigators independently reviewed responses, developed inductive codes, and grouped them into themes; discrepancies were resolved through discussion until consensus was achieved. Frequencies of each theme are reported as n (%) to provide additional context on the relative prevalence of each perspective among respondents.

### Evaluation Process at JHUSOM During the Implementation Phase From November 2024 to June 2025

By November 2024, the NeuroBricks Library had expanded to 13 modules. Students were then required to complete 6 NeuroBricks of their choosing, and the evaluation survey was revised to align with core clerkship learning objectives. Between November 2024 and June 2025, students responded to key statements using a 5-point Likert scale (1 = “strongly disagree”; 5 = “strongly agree”), assessing perceived impact of NeuroBricks on symptom recognition, examination interpretation, readiness for clinical encounters, and learning preferences. Students were also asked whether the informational content of the NeuroBricks was “not enough,” “just right,” or “too much” for their stage of training. Responses were summarized using descriptive statistics (counts and percentages) to assess perceived value and utility.

### Perceived Value of the NeuroBrick Construction Process and Global Engagement With the NeuroBricks Library

Additional insights into NeuroBrick developers' perceptions of the creation process were obtained through an informal, anonymous survey. The survey consisted primarily of items rated on a 5-point Likert scale (from “strongly disagree” to “strongly agree”), along with 1 multiple-response item asking participants to select all perceived benefits of their participation. The survey was distributed to undergraduate students, medical students, residents, fellows, and faculty involved in NeuroBricks development. Items explored reflections on the value of the experience, perceived learning gains, mentorship, collaboration, and sense of community. Respondents identified their role in the process, but no other identifying information was collected. In addition, the ScholarRx platform provided anonymized data on the global reach of the library by location and number of users (students and faculty).

### Standard Protocols Approvals, Registrations, and Participant Consents

This study was reviewed and deemed exempt by the JHUSOM Institutional Review Board (IRB). As such, the need for informed consent was waived (IRB00337604).

### Data Availability

Anonymized data not included in this publication are available on reasonable request from qualified investigators and with IRB approval.

## Results

### JHUSOM Results

“I Have a Tremor,” the first module developed for the NeuroBricks Library, was evaluated by 252 second-year, third-year, and fourth-year medical students at JHUSOM between January 3, 2023, and October 10, 2024. On a 5-point Likert scale (1 = strongly disagree; 5 = strongly agree), the module received a mean satisfaction rating of 4.08 (SD = 0.94) (Table 1).

**Table 1:** Mean Student Satisfaction Ratings for Individual Pilot NeuroBricks

Pilot NeuroBricks	Time span	Mean	SD	Number of students
“I Have a Tremor”	January 2023–October 2024	4.08	0.94	252
“I Can't See Out of My Eye”	November 2023–October 2024	4.10	0.93	97
“I Can't Walk, and I Can't Feel My Legs!”	November 2023–October 2024	4.11	0.91	97
“I'm Feeling Off Balance”	November 2023–October 2024	4.10	0.93	97
“My Vision Is Not Right, and My Arm Is Tingling”	November 2023–October 2024	4.09	0.94	97

Abbreviation: JHUSOM = Johns Hopkins University School of Medicine.

Student satisfaction ratings for 5 pilot clinical NeuroBricks developed at JHUSOM collected between January 2023 and October 2024. Ratings were measured on a 5-point Likert scale (1 = strongly disagree, 5 = strongly agree) across 97–252 respondents per Brick. All NeuroBricks received similarly high ratings, with mean scores ranging from 4.08 to 4.11.

Between November 2023 and October 2024, 97 medical students at JHUSOM evaluated 4 additional NeuroBrick modules. The mean (SD) satisfaction scores on a 5-point Likert scale for each module were as follows: “I Can't See Out of My Eye”, 4.10 (0.93); “I Can't Walk, and I Can't Feel My Legs!”, 4.11 (0.91); “I'm Feeling Off Balance”, 4.10 (0.93); and “My Vision Is Not Right, and My Arm Is Tingling”, 4.09 (0.94) (Table 1).

Most students (56 [57.7%]) agreed or strongly agreed that the NeuroBricks were helpful resources for the neurology clerkship, whereas 28 (28.9%) remained neutral and 13 (13.4%) disagreed or strongly disagreed. When asked whether they would recommend the resource to other students, 40 (41.2%) agreed or strongly agreed, 40 (41.2%) were neutral, and 17 (17.5%) disagreed or strongly disagreed. In addition, 93.8% of students agreed that the clerkship provided appropriate learning tools (including NeuroBricks) and opportunities to meet learning objectives (Table 2).

**Table 2:** Student Perceptions of the Pilot NeuroBricks at JHUSOM (N = 97)^a^

Survey item	Number of students	Percentages
The bricks were helpful as a resource for the neurology clerkship		
Agree/strongly agree	56	57.7
Neutral	28	28.9
Strongly disagree/disagree	13	13.4
I would recommend this resource to other students		
Agree/strongly agree	40	41.2
Neutral	40	41.2
Strongly disagree/disagree	17	17.5
The neurology clerkship provided appropriate learning tools or opportunities for me to attain the learning objectives		
Agree/strongly agree	91	93.8
Neutral	5	5.2
Strongly disagree/disagree	1	1.0

Abbreviation: JHUSOM = Johns Hopkins University School of Medicine.

Survey results show that 57.7% found the NeuroBricks helpful, and 41.2% would recommend them; a substantial portion remained neutral. In addition, 93.8% agreed that the clerkship provided appropriate tools or opportunities to meet learning objectives.

a Data collected between November 2023 and October 2024.

Between November 2024 and June 2025, 71 medical students at JHUSOM completed additional evaluation questions about the NeuroBricks Library, which included the 5 pilot NeuroBricks as well as 8 subsequently developed NeuroBricks. Most students (54 [76.1%]) agreed or strongly agreed that the NeuroBricks improved their recognition of neurologic symptoms and helped them prepare to see patients in a clinical setting (52 [73.2%]). Over two-thirds (48 [67.6%]) also reported improved interpretation of abnormal neurologic findings. Most preferred asynchronous learning (49 [69%]), and interactive features were rated as engaging by more than half of respondents (48 [67.6%]). Most medical students (64 [90.1%]) felt that the information provided by the NeuroBricks on various neurologic topics was appropriate to their stage in medical education training (Table 3).

**Table 3:** Perceptions of Expanded NeuroBricks Library at JHUSOM (N = 71)^a^

Survey item	Number of students	Percentages
The NeuroBricks improved my recognition of symptoms that may signify neurological diseases		
Agree/strongly agree	54	76.1
Neutral	10	14.1
Strongly disagree/disagree	7	9.9
The NeuroBricks improved my recognition and interpretation of abnormal findings on the neurological examination		
Agree/strongly agree	48	67.6
Neutral	15	21.1
Strongly disagree/disagree	8	11.3
The NeuroBricks were a helpful resource in preparing to see patients with neurological conditions		
Agree/strongly agree	52	73.2
Neutral	12	16.9
Strongly disagree/disagree	7	9.9
I prefer asynchronous self-paced learning (such as the Bricks) over in-person faculty lectures		
Agree/strongly agree	49	69.0
Neutral	9	12.7
Strongly disagree/disagree	13	18.3
The NeuroBricks are interactive with features (i.e. flashcards, embedded graphs, videos, etc) that enhance engagement		
Agree/strongly agree	48	67.6
Neutral	18	25.4
Strongly disagree/disagree	5	7.0
How much information do you feel the NeuroBricks provide you with on neurological topics at this stage of your medical education?		
Just Right	64	90.1
Too Much	1	1.4
Not Enough	6	8.5

Abbreviation: JHUSOM = Johns Hopkins University School of Medicine.

Students rated 6 statements on a 5-point Likert scale (1 = strongly disagree, 5 = strongly agree). Most students felt that the NeuroBricks improved symptom recognition (76.1%), examination interpretation (67.6%), and preparedness to see patients (73.2%). The majority also preferred asynchronous learning (69%) and found interactive features engaging (67.6%). Most (90.1%) reported the amount of information was appropriate for their level of training.

a Data collected between November 2024 and June 2025.

### HMS Results

Between May 2023 and January 2024, 20 of 45 second-year, third-year, and fourth-year medical students at HMS optionally completed and evaluated the 5 pilot NeuroBrick modules. Most students (17 [85%]) reported that they were likely or very likely to recommend the NeuroBricks to other students. Students reported using the NeuroBricks for various purposes during the clerkship, including as a general learning resource (8 [40%]), to study for the National Board of Medical Examiners (NBME) Subject Examination (9 [45%]), or only to meet requirements (8 [40%]) while 8 students (40%) also indicated use “in some other way” (Table 4).

**Table 4:** Study Practices and Perceptions of NeuroBricks at HMS (n = 20)^a^

Survey item	Number of students (%)
The bricks were helpful as a resource for the neurology clerkship	
Likely/very likely	17 (85)
Not too likely/not at all likely	3 (15)
How did you use the NeuroBricks to study during the clerkship? Check all that apply	
As a general learning resource for the clerkship	8 (40)
To Study for the NBME	9 (45)
Only for Requirements	8 (40)
In some other way	8 (40)
No Answer	8 (40)

Abbreviation: HMS = Harvard Medical School.

Survey findings reflect students' likelihood of recommending NeuroBricks, reported study practices, and qualitative impressions. Eighty-five percent were likely/very likely to recommend the resource. Students used the NeuroBricks for general learning, for NBME preparation, or to meet clerkship requirements.

a Data collected between May 2023 and June 2025.

Thematic analysis of student responses (n = 20) to open-ended survey questions revealed 4 key themes regarding the use and perceived impact of NeuroBricks. These included its use as a targeted or foundational learning tool, preference for other resources, and improved confidence or self-efficacy. A summary of these themes, including representative frequencies, is presented in Table 5.

**Table 5:** Thematic Analysis of Student Perceptions of NeuroBricks at HMS (n = 20)^a^

Theme	Number of responses (%)	Example quotes
Used as a targeted or in-depth resource	5 (25)	“I will review Bricks for any topic that I want more in-depth reading on.”
		“For future cases, when I feel as if I do not have a good background I will look to see if there is a module present for that topic.”
Use as a foundational or supplementary tool before practice questions	3 (15)	“I will use Rx Bricks if I want deeper knowledge on a topic that I am studying in First Aid.”
		“I will use it as content studying before doing practice questions.”
Preference for other resources	3 (15)	“I probably won't use it… seemed a bit too surface level to be helpful for the shelfs.”
Increased confidence and self-efficacy	7 (35)	“I felt more confident… on the rounds. I could anticipate the questions my attending/team might ask me about the patient I was caring for.”
		“…it helped me feel more confident putting together a cohesive understanding of a disease, building up from the pathophysiology.”
		“It helped me improve in what questions to ask patients and feel more comfortable with the diagnosis…”

Abbreviation: HMS = Harvard Medical School.

Thematic analysis of open-ended responses revealed 4 themes: targeted or in-depth use, foundational support before practice questions, concerns about access/cost or preference for other tools, and increased confidence and self-efficacy.

a Data collected between May 2023 and June 2025.

### Perception of the NeuroBrick Construction Process Results

The NeuroBrick construction process began with 4 neurology faculty members—three from Johns Hopkins and 1 from HMS. This initiative evolved into a vCOP using a formalized construction process and a multi-tiered mentorship model, which included 4 additional faculty members in editorial or expert content review roles, 3 fellows, 6 residents, 8 medical students, and 1 undergraduate student.

Of the 26 contributors to the 13 NeuroBricks, 13 (50%) voluntarily completed an informal, anonymous survey designed to provide initial feedback on the multi-tiered mentorship model used in the NeuroBrick construction process. Respondents included 5 faculty members, 5 residents or fellows, 2 medical students, and 1 undergraduate student.

All 5 faculty members indicated that participation was a worthwhile use of their time; 4 reported meaningful contributions to their professional development, and 4 noted that the process fostered a sense of community. Among residents and fellows, all 5 found the experience worthwhile, with 4 citing professional development, 3 noting a sense of community, and 4 highlighting that the process encouraged deeper exploration of concepts during feedback discussions. It is important to note that all 5 reported receiving meaningful guidance.

**Figure 2:**
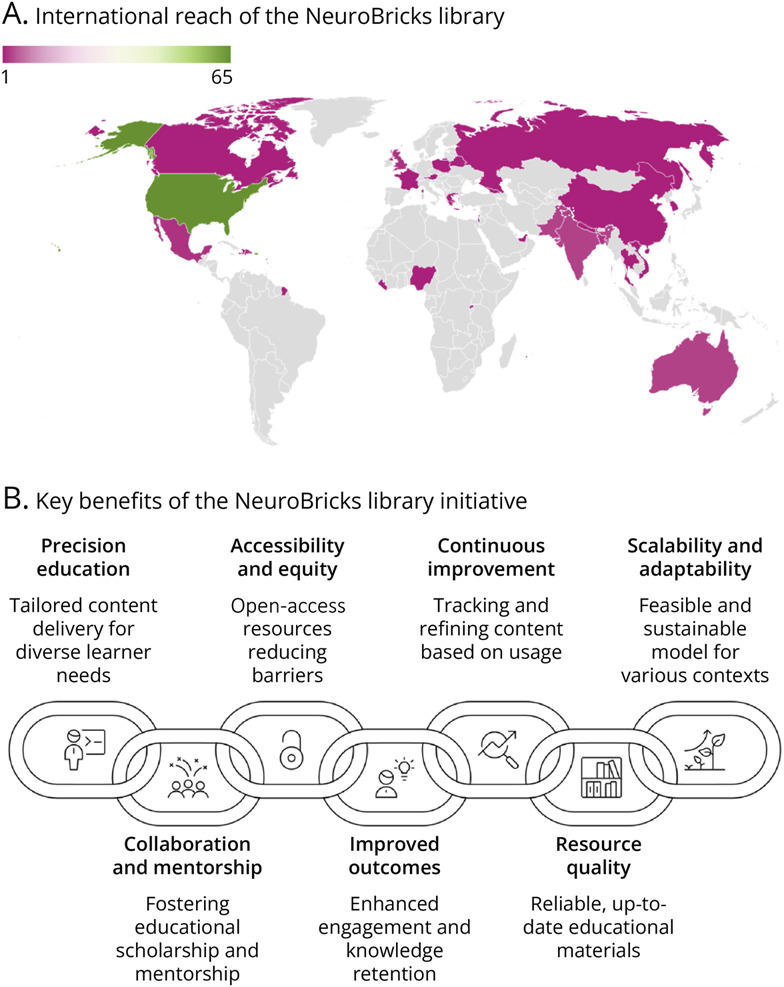
Feasibility and Impact of the NeuroBricks Library's Multi-Tiered Mentorship Model (A) Across the NeuroBricks Library, 817 students and 114 faculty members from 108 medical schools across 34 countries completed at least 1 module. (B) Key benefits of the NeuroBricks Library initiative: This collaborative, multi-tiered mentorship model for developing peer-reviewed, open-access neurology modules supports precision education, global accessibility, mentorship, learner engagement, and sustainability across disciplines. Key: darker shading on the map indicates higher user numbers per country.

All 3 student respondents likewise described the experience as worthwhile. Two reported gains in professional development, 2 felt more connected to a community, and all 3 emphasized that the process both encouraged critical exploration of concepts and provided meaningful guidance. Participants also identified specific benefits of joining this vCOP, as illustrated in Figure 1B. Anonymized platform-tracked data suggested that across the 13 NeuroBricks, 817 students and 114 faculty members from 108 medical schools across 34 countries have completed at least 1 module, although most of the end users were located in the United States (Figure 2A).

## Discussion

This feasibility study demonstrates that the NeuroBrick construction process is an efficient, effective, and sustainable model for creating and validating peer-reviewed online medical education modules for clerkship students. Through interinstitutional collaboration, this model supports the development of content that meets the learning needs of modern-day medical students. By embracing the principles of precision education, the model has the potential for tailored content delivery—adapting modules to specific curricula, learner levels, and institutional contexts, including settings where certain specialties, such as neurology, may not be formally taught (Figure 2).^23^

Since October 2024, additional modules have been developed as part of this initiative. As of June 2025, 13 NeuroBricks have been developed, with at least 2 more in progress. This effort leverages the NeuroBrick platform to advance what we have termed the “Three Cs” of virtual learning: (1) creating new educational content, (2) curating existing resources for diverse curricular needs, and (3) connecting educators and learners across institutional and geographic boundaries. Through this model, educators not only contribute to the expansion of digital learning but also share the workload of content creation and adaptation. In the postpandemic era, virtual collaboration has become not just more accessible but a powerful engine for cross-institutional innovation and global exchange.^24,25^ These virtual communities of practice, when built around a shared educational goal, have been shown to effectively support resource sharing, collaborative problem solving, and educational advancement across stakeholders, including learners, faculty, and institutions.^16,17,26^

The multi-tiered mentorship framework is a vCOP that leverages cost-effective, accessible technology to enable asynchronous collaboration among geographically dispersed faculty and trainees—many of whom might not otherwise have opportunities for co-development. This structure not only minimizes the time required to create high-quality resources but also promotes educational scholarship and cultivates the development of meaningful mentor-mentee relationships. In addition, it introduces medical students, residents, and fellows to potential career paths in medical education. As illustrated in Figure 1, NeuroBrick developers reported gains such as mentorship, professional development, and community building, supporting the feasibility and impact of the model.

Medical educators at our institutions have also incorporated NeuroBricks into flipped classroom teaching, reducing the need for time-intensive slide-based lectures and drawing instead on a library of high-quality, premade resources that enhance engagement and learning outcomes.^27,28^ Students, in turn, can review modules in preparation for bedside teaching or clinical encounters. It is important to note that this initiative aligns with the preferences of today's digital-native learners, who value concise, interactive, and immediately applicable content.

Before implementing the NeuroBricks Library, we have observed neurology clerkship students relying on widely circulated but inaccurate resources to their detriment, leading to confusion about neurologic concepts. This challenge is likely amplified at institutions without required neurology clerkships, where students often lack expert guidance in evaluating online materials. NeuroBricks address these gaps by providing peer-reviewed, evidence-based content that is continuously updated to reflect current knowledge. Its open-access model, supported by Creative Commons licensing, lowers financial and institutional barriers while promoting equity in global medical education. Platform analytics further allow educators to track reach, usage, and engagement, enabling iterative, data-driven improvements based on real-world engagement. As shown in Figure 2A, the NeuroBricks Library has been accessed by over 900 learners and faculty from 108 medical schools across 34 countries, providing concrete evidence of its global scalability and impact.

Together, these features position the NeuroBricks Library as a sustainable, adaptable, and widely applicable resource for advancing clinical education across diverse institutional contexts.

While our findings indicate a positive reception from students who used the NeuroBricks, several limitations should be acknowledged. Because the survey items were not derived from validated instruments, psychometric properties such as reliability and validity were not assessed; this is a common limitation in exploratory educational evaluations and should be considered when interpreting the results. It is also worth noting that the high satisfaction ratings among HMS users likely reflect significant selection bias, as those who chose to engage with the optional resource may have been predisposed to favor this learning format or had learning needs particularly well matched to the NeuroBricks' approach.

While the NeuroBricks Library offers a promising solution to the challenges posed by inconsistent online content, our evaluation revealed that its impact depends heavily on how it is integrated within institutional curricula. At JHUSOM, where the NeuroBricks replaced traditional clerkship didactics as a required component, student engagement was substantially higher than at HMS, where the resource was offered as optional supplementary material. This disparity highlights several important considerations for educational resource implementation. First, the dramatically lower usage among HMS students suggests that simply making resources available without formal integration into the curriculum may limit their reach. In today's educational environment, medical students are inundated with study materials and must continuously prioritize their limited time and attention. As a result, even high-quality optional resources may be overlooked in favor of tools perceived to be directly aligned with examination preparation. In particular, medical students often prioritize studying for shelf examinations and USMLE content, which may not align with the clinical, patient-centered learning emphasized in the clerkship curriculum. This observation underscores the potential value of providing clearer guidance to students regarding recommended resources rather than expanding the array of available options. Finally, although this initiative used the ScholarRx platform, we did not compare the development process or user experience with other authoring platforms. Similar approaches could likely be implemented using alternative digital tools with comparable functionality, although it remains uncertain whether other platforms provide the same combination of features, such as cloning, multiauthor collaboration, versioning with attribution, integrated assessments, and Creative Commons license support.

Future directions include expanding partnerships with US and international medical schools to build a truly global community of practice. Many medical students in both low-income countries and high-income countries experience “neurophobia”—a fear or discomfort with the neurosciences stemming from the perceived complexity of neurology and inconsistent access to educational resources and clinical training.^29-32^ Contributing factors include the difficulty of neuroanatomy, limited exposure to neurologic cases, and the perceived challenge of making neurologic diagnoses.^30^ The lack of dedicated neurology clerkships at many institutions further exacerbates the problem. By providing vetted, adaptable, high-quality modules, the initiative aims to support learners globally and help address the worldwide burden of neurologic disease, which remains the leading cause of disability.^1,28^ Although developed within the field of neurology, this open-source model can be adapted across various disciplines, with particular urgency in specialties where educational disparities and workforce shortages are most severe.

The NeuroBricks Library initiative represents a sustainable, scalable model for developing high-quality, peer-reviewed, and globally accessible educational content. By aligning modern pedagogical approaches with collaborative digital tools, this initiative not only addresses critical gaps in neurology education but also establishes a foundation for broader impact across medical disciplines and international borders.

## Glossary

HMS: Harvard Medical School
IRB: Institutional Review Board
JHUSOM: Johns Hopkins University School of Medicine
vCoP: virtual community of practice
